# *miR‐100* rs1834306 a > G polymorphism decreases neuroblastoma risk in Chinese children

**DOI:** 10.1002/cnr2.1875

**Published:** 2023-07-28

**Authors:** Yufeng Han, Jiaming Chang, Lei Lin, Chunlei Zhou, Jinhong Zhu, Haiyan Wu, Jing He, Wen Fu

**Affiliations:** ^1^ Department of Pediatric Surgery Guangzhou Institute of Pediatrics, Guangdong Provincial Key Laboratory of Research in Structural Birth Defect Disease, Guangzhou Women and Children's Medical Center, Guangzhou Medical University, Guangdong Provincial Clinical Research Center for Child Health Guangzhou Guangdong China; ^2^ Department of Pathology Children's Hospital of Nanjing Medical University Nanjing Jiangsu China; ^3^ Department of Clinical Laboratory, Biobank Harbin Medical University Cancer Hospital Harbin Heilongjiang China

**Keywords:** *miR‐100*, neuroblastoma, polymorphism, susceptibility

## Abstract

**Background:**

Neuroblastoma is a common malignant tumor stemming from the sympathetic nervous system in children, which is often life‐threatening. The genetics of neuroblastoma remains unclear. Studies have shown that miRNAs participate in the regulation of a broad spectrum of biological pathways. The abnormity in the miRNA is associated with the risk of various cancers, including neuroblastoma. However, research on the relationship of miRNA polymorphisms with neuroblastoma susceptibility is still in the initial stage.

**Methods:**

In this research, a retrospective case–control study was conducted to explore whether *miR‐100* rs1834306 A > G polymorphism is associated with neuroblastoma susceptibility. We enrolled 402 cases and 473 controls for the study. The logistic regression analysis was adopted to calculate odds ratios (ORs) and 95% confidence intervals (CIs) for the association between *miR‐100* rs1834306 A > G and neuroblastoma risk.

**Results:**

Our results elucidated that the *miR‐100* rs1834306 A > G polymorphism was associated with the decreased risk of neuroblastoma (AG versus AA: adjusted OR = 0.72, 95% CI = 0.53–0.98, and *P* = 0.038). The subsequent stratified analysis further found that rs1834306 AG/GG genotype reduced the risk of neuroblastoma in the subgroup with tumors of the mediastinum origin (adjusted OR = 0.63, 95% CI = 0.41–0.95, and *P* = 0.029).

**Conclusions:**

In summary, *miR‐100* rs1834306 A > G polymorphism was shown to associate with decreased neuroblastoma risk in Chinese children, especially for neuroblastoma of mediastinum origin. This conclusion needs to be verified in additional large‐size case–control studies.

## 1 INTRODUCTION

Neuroblastoma is the most frequently encountered extracranial solid tumor in childhood, most of which occurs in infancy.^1^ This tumor primarily originates from adrenal and paraspinal sympathetic nerve chains, and ranks fourth among pediatric tumors. According to the reports, the annual incidence rate of neuroblastoma is as high as 11 cases per million among children aged 0–5 in France, Israel, Switzerland, and other high‐incidence areas.^2^ At the same time, the disease attacks 25 and less than 5 children per million in America and India, respectively.^3^ In China, its incidence rate is around 7.7 in a million children.^4^ Research shows that parents' long‐term exposure to wood dust, solder, radiation, and diesel fuel increases the risk of neuroblastoma in descendants.^5^, ^6^

In the previous studies, genomic amplification of *MYCN* was reported to have a vital role in the initiation and development of neuroblastoma.^7^, ^8^ The first genome‐wide association study (GWAS) found that the risk of neuroblastoma in Caucasian populations is significantly associated with three *CASC15* gene polymorphisms (rs6939340 A > G, rs4712653 T > C, and rs9295536 C > A) in the 6p22 region.^9^ The association was confirmed in the validation study conducted by our research team.^10^ Other than those susceptibility loci, more and more neuroblastoma‐associated single nucleotide polymorphisms (SNPs) have been discovered, including *TGFBR3L*,^11^ *BARD1*,^12^ *LMO1*,^13^ *HACE1*, *LIN28B*.^14^ However, the known SNPs explain only part of the etiology of neuroblastoma, and more causal genes SNPs need to be ulteriorly identified.

miRNAs are referred to as small non‐coding RNAs composed of about 22 nucleotides, which control the expression of about 30% of genes in the human genome.^15^ Numerous studies have shown generally dysregulated miRNA expression levels in tumors compared with normal tissues.^16^ Although there has been evidence that abnormal expression of miRNA genes may account for carcinogenesis,^17^, ^18^, ^19^, ^20^ the association of miRNA polymorphisms with neuroblastoma susceptibility remains to be investigated. The previous two‐center case–control study demonstrated that *miR‐34b/c* rs4938723 T > C polymorphism and *miR‐218* rs11134527 A > G polymorphism conferred neuroblastoma susceptibility.^21^ Studies indicated that most miRNA loci are located at “fragile sites” on chromosomes.^22^ These sites are susceptible to tumorigenic mutations. The genetic association between miRNA SNPs and cancer susceptibility is tissue‐specific.^23^ Deletion or amplification of specific miRNAs can lead to the promotion or suppression of carcinogenesis.^24^ miR‐100 belongs to the miR‐99 family and its dysregulation has been observed in various cancer types.^25^ Abnormal expression of *miR‐100* plays a crucial role in cancer through a complex regulatory network.^26^ Therefore, understanding *miR‐100* polymorphism may greatly facilitate cancer diagnosis, treatment, and prognosis. So far, researches on the relationship between *miR‐100* SNPs and neuroblastoma predisposition are still lacking. Herein, we attempted to determine the effects of *miR‐100* rs1834306 A > G variant on neuroblastoma susceptibility.

## 2 MATERIALS AND METHODS

### 2.1 Study population

In this study, we recruited 402 neuroblastoma patients and 473 cancer‐free controls from Jiangsu Province (Table S1).^27^ The neuroblastoma patients were selected based on criteria that are confirmed by biopsy or histology. Moreover, the age and gender distributions of cases and cancer‐free controls were matched. We classified the patients into five stages and an invalid group following International Neuroblastoma Staging System (INSS).^28^ Meanwhile, neuroblastoma cases were also classified based on various sites of origin. Each subject or his/her guardian has signed informed written consent, and this research was also approved by the Institutional Review Board of Children's Hospital of Nanjing Medical University (Approval No: 202112141–1).

### 2.2 SNP selection and genotyping

SNPs were screened from the dbSNP database and SNPinfo website. We selected the *miR‐100* rs1834306 A > G for the current study.^29^ The peripheral blood samples were processed to obtain genomic DNA with the utilization of the TIANamp Blood DNA Kit (TianGen Biotech Co. Ltd., Beijing, China).^10^ The purity and concentration of the extracted DNA were further measured by using UV spectrophotometer (Nano Drop Technologies, Inc., Wilmington, DE). We used Taqman real‐time PCR method (Applied Biosystems, CA, USA) to determine the SNP genotype in samples, which has been mentioned previously.^30^, ^31^, ^32^ Regarding quality control, 10% of the genotyping samples were randomly retested, and a genotype consensus rate of 100% was attained.

### 2.3 Statistical analysis

We performed a Chi‐square test to determine the differences in demographic characteristics (age and gender) and genotype distributions between cases and controls. We evaluated whether the departure of the selected polymorphisms in the controls violated the Hardy–Weinberg equilibrium (HWE) with a goodness‐of‐fit χ^2^ test. In addition, we used the logistic regression analysis to obtain odds ratios (ORs) and 95% confidence intervals (CIs) for the association between *miR‐100* rs1834306 A > G and neuroblastoma risk. Then, a stratification analysis was performed according to age, gender, sites of tumor origin, and clinical stages to assess further the association of *miR‐100* rs1834306 A > G polymorphism with neuroblastoma risk. The *P* value is effective when it is lower than 0.05. All statistics were performed by SAS software (v10.0 SAS Institute Inc., Cary, NC).

## 3 RESULTS

### 3.1 Association of the SNP *miR* *‐100* rs1834306 A > G with neuroblastoma risk

The association of the rs1834306 A > G with neuroblastoma risk is displayed in Table 1. The *P*‐value of HWE for rs1834306 A > G in the controls was 0.566, suggesting the genotype distribution of the SNP did not violate HWE. We discovered that the rs1834306 AG genotype polymorphism was related to a decreased risk of neuroblastoma (adjusted OR = 0.72, 95% CI = 0.53–0.98, and *P* = 0.038) with the AA genotype as a reference group.

**TABLE 1:** *miR‐100* rs1834306 A > G polymorphism and neuroblastoma risk in children from Jiangsu province.

Genotype	Cases (*N* = 401)	Controls (*N* = 473)	*P* ^a^	Crude OR (95% CI)	*P*	Adjusted OR (95% CI)^b^	*P* ^b^
rs1834306 (HWE = 0.566)							
AA	135 (33.67)	136 (28.75)		1.00		1.00	
AG	173 (43.14)	241 (50.95)		**0.72 (0.53–0.98)**	0.039*	**0.72 (0.53–0.98)**	0.038*
GG	93 (23.19)	96 (20.30)		0.98 (0.67–1.42)	0.898	0.98 (0.67–1.41)	0.893
Additive			0.680	0.96 (0.80–1.16)	0.680	0.96 (0.80–1.16)	0.679
Dominant	266 (66.33)	337 (71.25)	0.118	0.80 (0.60–1.06)	0.118	0.79 (0.60–1.06)	0.116
AA/AG	308 (76.81)	377 (79.70)		1.00		1.00	
GG	93 (23.19)	96 (20.30)	0.300	1.19 (0.86–1.64)	0.300	1.19 (0.86–1.64)	0.299

*Note*: *, Values were in bold if the 95% CIs excluding 1.00 or the *P* values less than 0.05.

Abbreviations: CI, confidence interval; OR, odds ratio; HWE, Hardy–Weinberg equilibrium.

^a^ χ^2^ test for genotype distributions between neuroblastoma cases and cancer‐free controls.

^b^ Adjusted for age and gender.

### 3.2 Stratification analysis

We further evaluated the association of *miR‐100* rs1834306 A > G polymorphism with neuroblastoma risk in different strata using stratification analysis (Table 2). Multiple subgroups were classified according to age, gender, sites of origin, and clinical stages. Interestingly, rs1834306 AG/GG genotypes exerted a risk reduction effect against neuroblastoma only in subgroups with neuroblastomas in the mediastinum (adjusted OR = 0.63, 95% CI = 0.41–0.95, and *P* = 0.029). The finding indicates that the impact of this SNP on neuroblastoma risk may be tissue‐specific.

**TABLE 2:** Stratification analysis for the association between *miR‐100* rs1834306 A > G polymorphism and neuroblastoma susceptibility.

Variables	rs1834306 (cases/controls)		OR (95% CI)	*P*	AOR (95% CI)^a^	*P* ^a^
	AA	AG/GG				
Age, month						
≤18	48/37	90/102	0.68 (0.41–1.14)	0.142	0.68 (0.41–1.14)	0.142
>18	87/99	176/235	0.85 (0.60–1.21)	0.368	0.85 (0.60–1.21)	0.363
Gender						
Females	56/58	135/167	0.84 (0.54–1.29)	0.420	0.84 (0.54–1.29)	0.420
Males	79/78	131/170	0.76 (0.52–1.12)	0.166	0.76 (0.52–1.12)	0.163
Sites of origin						
Adrenal gland	25/136	68/337	1.10 (0.67–1.81)	0.715	1.11 (0.67–1.82)	0.695
Retroperitoneal	59/136	107/337	0.73 (0.50–1.07)	0.103	0.73 (0.50–1.06)	0.099
Mediastinum	47/136	73/337	**0.63 (0.41–0.95)**	0.028*	**0.63 (0.41–0.95)**	0.029*
Others	2/136	16/337	3.23 (0.73–14.23)	0.122	3.21 (0.73–14.15)	0.124
Clinical stages						
I+II+4s	53/136	120/337	0.91 (0.63–1.34)	0.641	0.90 (0.62–1.32)	0.599
III+IV	53/136	110/337	0.84 (0.57–1.23)	0.365	0.84 (0.57–1.23)	0.374

*Note*: *, Values were in bold if the 95% CIs excluding 1.00 or the *P* values less than 0.05.

Abbreviations: AOR, adjusted odds ratio; CI, confidence interval; OR, odds ratio.

^a^ Adjusted for age and gender, omitting the corresponding stratify factor.

## 4 DISCUSSION

Mutations in the genetic genome often affect susceptibility to pediatric cancers. The present study revealed that *miR‐100* rs1834306 A > G polymorphism conferred reduced neuroblastoma risk. The results provided additional evidence for the necessary implications of the genetic variations in miRNA on the neuroblastoma pathogenesis.

miRNAs can regulate mRNA expression, thereby regulating the cell cycle, apoptosis, and carcinogenesis.^33^ The SNPs of miRNAs can influence tumor susceptibility by influencing the biogenesis, maturation, or function of miRNAs.^34^ The chromosome region of *miRNA‐100* is situated at 11q24.1.^35^ miRNA‐100 is formed after multistep processing of long primary miRNA which is generated in the nucleus by RNA Polymerase II.^36^, ^37^ According to the reports, miR‐100 is integrated into a multi‐protein complex during its formation, and then miR‐100 binds to the target gene's mRNA with the assistance of the RNA‐induced silencing complex, eventually inhibiting the translation or inducing degradation of mRNAs.^35^, ^38^, ^39^ In recent years, miR‐100 has been shown to target many biomolecules that play important roles in carcinogenesis, thereby affecting carcinogenesis. Liu et al. indicated that miR‐100 could affect the growth, cell cycle, and apoptosis of cancer cells by regulating the post‐transcriptional expression of *PLK1* in non‐small cell lung cancer.^40^ Therefore, it is reasonable to speculate that miRNA‐100 may affect the occurrence of neuroblastoma in children through the regulation of target mRNA expression. However, further researches are needed to confirm this hypothesis.

Recent reports, *miR‐100* genetic abnormalities have been involved in the initiation and progression of tumors.^26^ As reported in the previous studies, miR‐100 can be a tumor‐promoting or ‐suppressing gene in different tumor types and microenvironments.^41^ Therefore, dysregulation of *miR‐100* expression may be related to various cancers. miR‐100 expression levels have been shown to decrease in some tumors, such as nasopharyngeal carcinoma^42^ and hepatocellular carcinoma (HCC),^43^ whereas it was found to increase in others, such as HCC^44^ and small cell lung cancer.^45^ Nevertheless, the abundance of miR‐100 may be different from the same type of cancer. According to the different disease types, regions and ethnicities, polymorphisms may have differential genetic effects on disease. Many previous studies have investigated the correlation between *miR‐100* rs1834306 polymorphism and tumor susceptibility. In 2015, Zhu et al.^46^ indicated that *miR‐100* rs1834306 might decrease the risk of esophageal squamous cell carcinoma. Later on, Chang et al.^47^ found that the rs1834306 may be involved in the increased risk of endometriosis. Zhu et al.^48^ reported that the *miR‐100* rs1834306 A > G polymorphism is associated with enhanced risk of Hirschsprung disease in children in southern China. Recently, Chang et al.^49^ discovered that the *miR‐100* rs1834306 A > G polymorphism might also increase predisposition to biliary atresia in Chinese children.

Here, our case–control retrospective study found the association of *miR‐100* rs1834306 A > G polymorphism with reduced neuroblastoma susceptibility, suggesting a significant effect of rs1834306 AG/GG genotype in reducing the risk of neuroblastoma of mediastinum origin. However, due to the limitations of multiple factors, further experiments are needed to confirm the results.

Moreover, some study limitations need to be noted. Firstly, the sample size was not large enough for this study, including only 402 cases and 473 cancer‐free controls, which may limit statistical power. Secondly, since the patients involved in the study were all Chinese, and the samples were all from Jiangsu Province, the study may be subjected to selection bias. Thirdly, we only selected one polymorphism for the study. More SNPs need to be investigated concerning neuroblastoma susceptibility in different ethnic groups to yield more reliable conclusions. Besides, we could not rule out the impact of environmental factors on the risk of neuroblastoma.

## 5 CONCLUSION

In conclusion, we found that *miR‐100* rs1834306 A > G polymorphism is associated with decreased neuroblastoma risk in Chinese children. This association is significant in neuroblastoma of mediastinum origin. Furthermore, more extensive and diverse population samples must be analyzed to verify our conclusions.

## AUTHOR CONTRIBUTIONS

**Yufeng Han:** Investigation (equal); writing – original draft (equal); writing – review and editing (equal). **Jiaming Chang:** Investigation (equal); writing – original draft (equal). **Lei Lin:** Investigation (equal); methodology (equal); writing – original draft (equal); writing – review and editing (equal). **Chunlei Zhou:** Funding acquisition (equal); investigation (equal); resources (equal); writing – review and editing (equal). **Jin‐Hong Zhu:** Investigation (equal); writing – review and editing (equal). **Haiyan Wu:** Investigation (equal); resources (equal); supervision (equal); writing – review and editing (equal). **Jing He:** Conceptualization (equal); data curation (equal); formal analysis (equal); funding acquisition (equal); investigation (equal); methodology (equal); supervision (equal); writing – review and editing (equal). **Wen Fu:** Conceptualization (equal); investigation (equal); supervision (equal); writing – review and editing (equal).

## CONFLICT OF INTEREST STATEMENT

The authors declare no conflict of interest.

## ETHICS STATEMENT

All participants or their guardians have signed informed written consent, and all studies were also approved by the Institutional Review Board of Children's Hospital of Nanjing Medical University (Approval No: 202112141‐1).

## Supporting information


**Table S1.** Demographic characteristics of neuroblastoma patients and cancer‐free controls from Jiangsu province

## Data Availability

All the data were available upon request.
