# Supplementary material for: miR‐100 rs1834306 a > G polymorphism decreases neuroblastoma risk in Chinese children
Source: Cancer Rep (Hoboken). 2023 Jul 28;6(10):e1875. doi: 10.1002/cnr2.1875 (PMC10598254; doi:10.1002/cnr2.1875)
Supplement: Supplementary file 1 — Table S1. Demographic characteristics of neuroblastoma patients and cancer‐free controls from Jiangsu province [file CNR2-6-e1875-s001.doc]

| **Table S1**. Demographic characteristics of neuroblastoma patients and cancer-free controls from Jiangsu province | | | | | |
| --- | --- | --- | --- | --- | --- |
| Variables | Cases (N=402) | | Controls (N=473) | | *P* ^a^ |
|  | No. | % | No. | % |  |
| Age range, month | 0.033-168.00 | | 0.367-168.00 | |  |
| Mean ± SD | 40.99 ± 35.49 | | 40.88 ± 29.76 | | 0.962 ^b^ |
| Age |  | |  | | 0.100 |
| ≤18 months | 139 | 34.58 | 139 | 29.39 |  |
| >18 months | 263 | 65.42 | 334 | 70.61 |  |
| Gender |  |  |  |  | 0.987 |
| Female | 191 | 47.51 | 225 | 47.57 |  |
| Male | 211 | 52.49 | 248 | 52.43 |  |
| Sites of origin |  |  |  |  |  |
| Adrenal gland | 93 | 23.13 | / | / |  |
| Retroperitoneal region | 167 | 41.54 | / | / |  |
| Mediastinum | 120 | 29.85 | / | / |  |
| Other region | 18 | 4.48 | / | / |  |
| NA | 4 | 1.00 | / | / |  |
| INSS stages |  |  |  |  |  |
| I | 108 | 26.87 | / | / |  |
| II | 63 | 15.67 | / | / |  |
| III | 59 | 14.68 | / | / |  |
| IV | 104 | 25.87 | / | / |  |
| 4s | 2 | 0.50 | / | / |  |
| NA | 66 | 16.42 | / | / |  |
| SD, standard deviation; NA, not available.  ^a^ Two-sided χ^2^ test between neuroblastoma patients and cancer-free controls.  ^b^ t test between neuroblastoma patients and cancer-free controls. | | | | | |
